# A Mysterious Case of an Infarcted Spleen due to Kissing Disease: A Rare Entity

**DOI:** 10.7759/cureus.6700

**Published:** 2020-01-19

**Authors:** Hira Pervez, Asim Tameez Ud Din, Ahmad Khan

**Affiliations:** 1 Internal Medicine/Cardiology, Dow University of Health Sciences, Karachi, PAK; 2 Internal Medicine, Rawalpindi Medical University, Rawalpindi, PAK; 3 Internal Medicine, West Virginia University, Charelston, USA

**Keywords:** ebv, infectious mononucleosis, splenic infarction, im, epstein-barr virus

## Abstract

Viruses are long known to be leading causes of self-limiting infections. Infectious mononucleosis (IM) caused by the Epstein-Barr virus (EBV) is, however, no exception. The ailment is caused by a DNA virus belonging to the Herpesviridae family. As stated earlier, the infection is usually self-limited with mononucleosis-like symptoms such as fever, sore throat, lymphadenopathy (LAD), rash, headache, etc. In rare instances, it can lead to severe complications. The organ of prime importance following the infection is spleen. There are occasions where splenic injuries can lead to rupture, deeming to emergency surgical interventions. At other times, a rare entity may also be seen that constitutes an infarction within the splenic substance. We present this rare finding in a 20-year-old male patient with a left upper quadrant (LUQ) pain, cervical LAD, and sore throat who was brought to the emergency department. On physical examination, mild tenderness was observed in the LUQ with an inflamed throat and palpable cervical and occipital lymph nodes. Laboratory investigations suggested lymphocytosis with no blast cells, lactic acidosis, and mild acute kidney injury. A contrast-enhanced computed tomography scan demonstrated an enlarged spleen with wedge-shaped hypodense areas, which led to a diagnosis of splenic infarction secondary to infectious mononucleosis. Keeping in mind the symptomatology and the age of the patient, a bunch of differentials were needed to be ruled out. Out of a series of investigations done on the patient, EBV serology for IgM was positive. The patient was conservatively treated with a complete resolution of symptoms in one month. Our case adds to the literature the finding of a rare etiology of splenic infarction secondary to IM and the importance of stepwise and cost-effective investigations to avoid unnecessary workup when needed.

## Introduction

Infectious mononucleosis (IM) is a fairly common condition mostly affecting the young, fertile population between 15 and 25 years of age. Its incidence is estimated to be approximately 45/100,000 within a population [1]. The etiological agent implicated in its pathogenesis is Epstein-Barr virus (EBV), with an oro-oral mode of spread. It presents with mononucleosis-like symptoms including sore throat, fever, lymphadenopathy (LAD), malaise, fatigue, etc. In the majority of cases, it is a self-limiting illness without any long-term sequelae. The other subset of patients can have dire complications. This can be due to the involvement of the reticuloendothelial system, such as the spleen, and can lead to either infarction or rupture [2]. Splenic infarction (SI) associated with IM is a rare entity. A total of 19 cases were highlighted in a literature review conducted between 1961 and 2015 [3,4]. Most of the observed cases of SI are seen in the pediatric population or patients with other hematological disorders. Here, we present a 20-year-old male who developed SI following IM and was conservatively treated with no late complications.

## Case presentation

**Figure 1:**
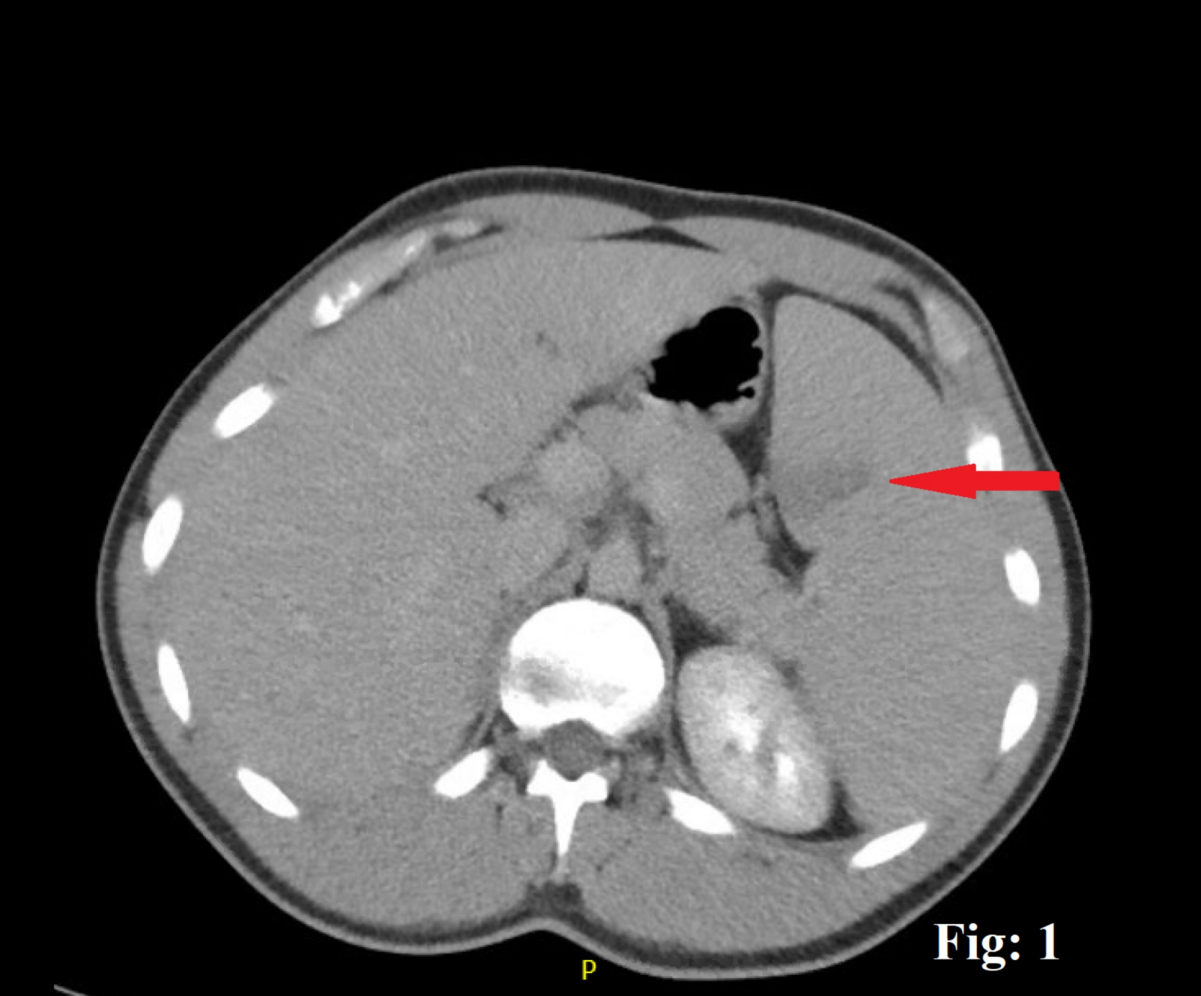
A contrast-enhanced computed tomograpghy scan of the abdomen, the red arrow showing wedge-shaped splenic infarct.

**Figure 2:**
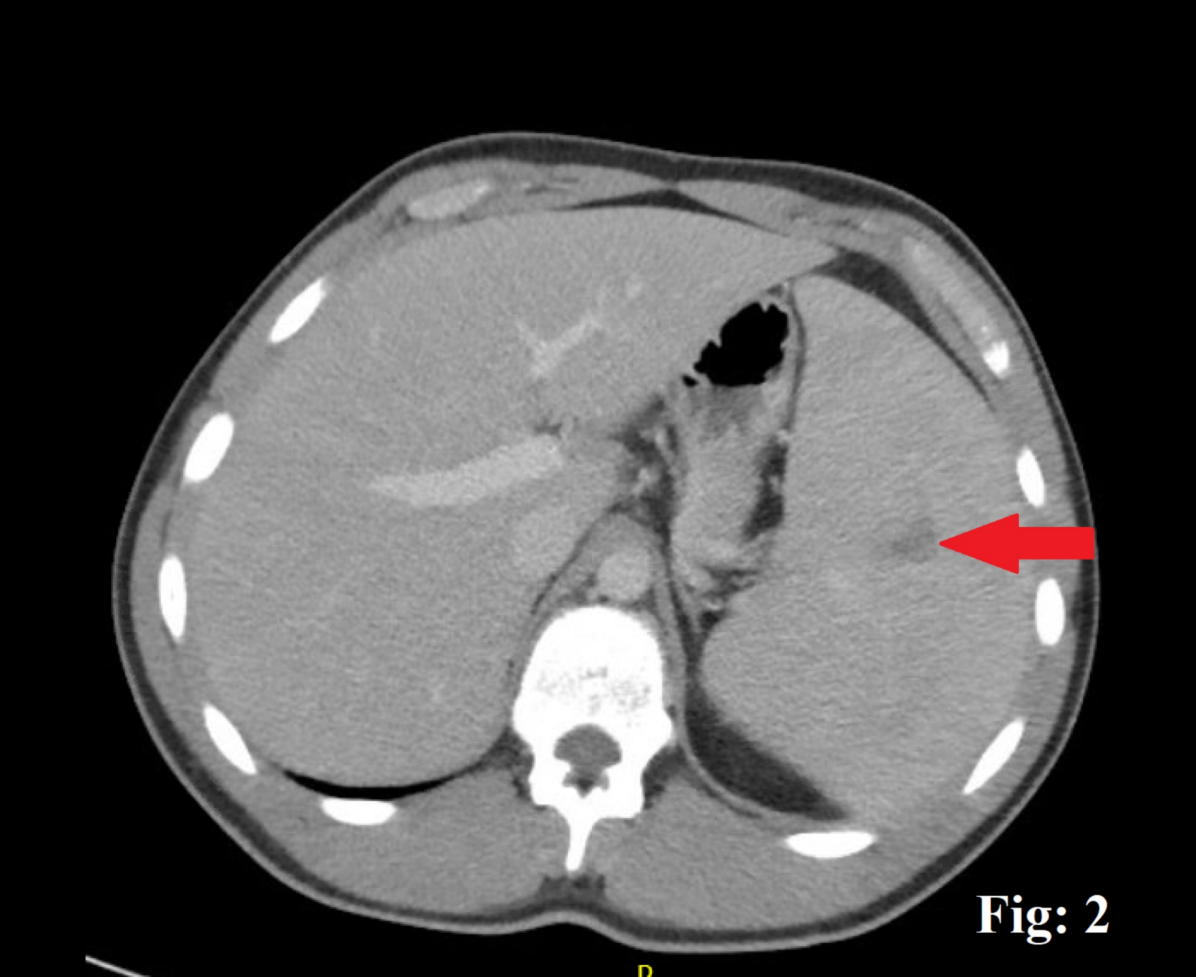
A contrast-enhanced computed tomography scan of the abdomen, the red arrow pointing wedge-shaped splenic infarct.

**Figure 3:**
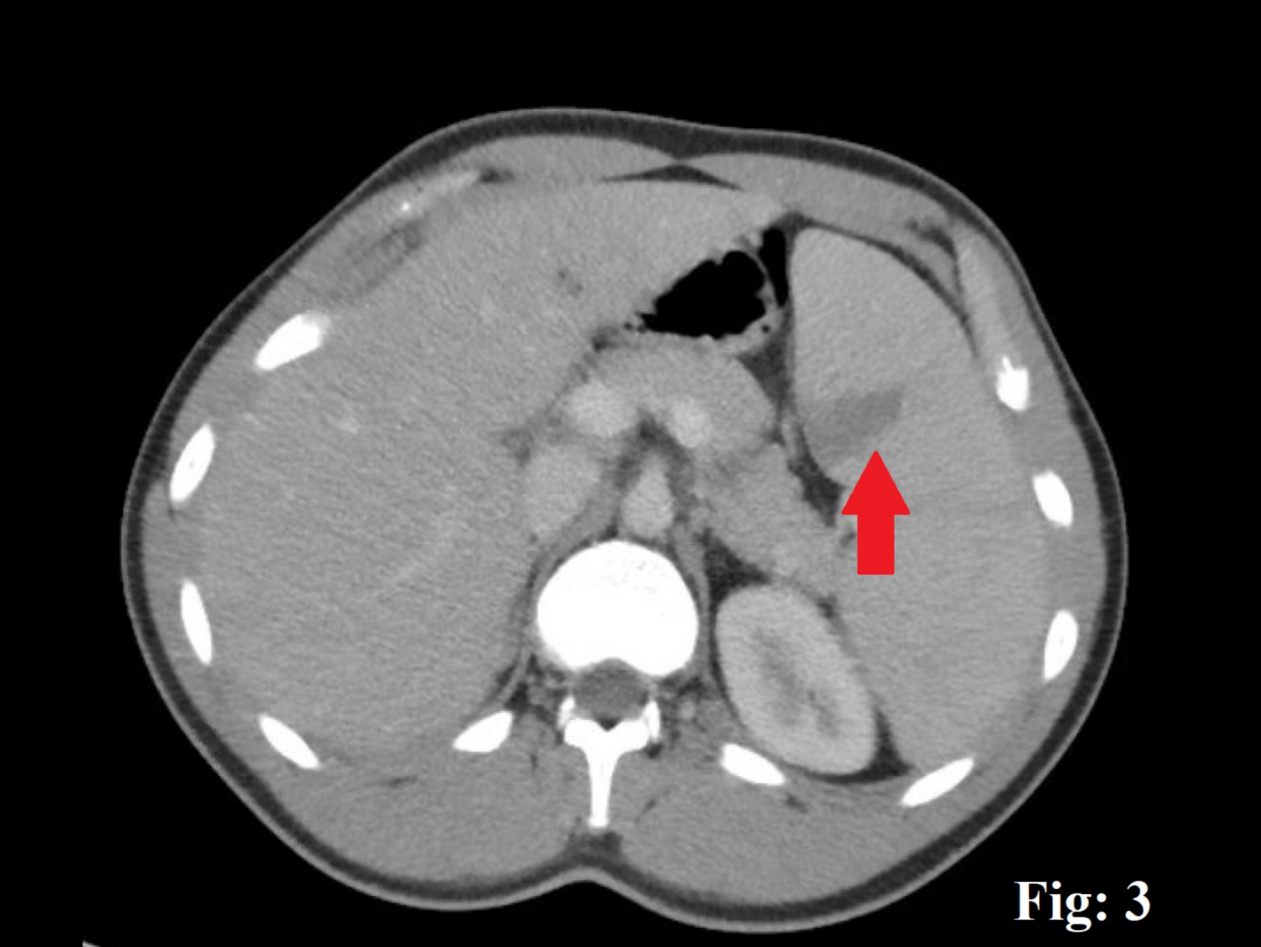
A contrast-enhanced computed tomography scan of the abdomen, the red arrow pointing a wedge-shaped splenic infarct.

A 20-year-old male with no significant comorbid conditions presented to the emergency department (ED) with a five-day history of a generalized, constant, crampy abdominal pain directed more toward the left upper quadrant (LUQ) without any aggravating or relieving factors. The pain was accompanied by a single episode of clear, non-bloody vomitus associated with nausea and inadequate food intake. A detailed review of his systems demonstrated a generalized malaise, myalgia, and a reduced appetite. The patient admits having a sore throat, subjective low-grade fever, and drenching night sweats for a few days. He denied any intentional or unintentional weight loss or noticing any lumps or bumps in his body. He denied any recent sexual or sick contact, travel history, smoking, alcohol, use of any illicit drugs, or a family history of cancers or hematological disorders. His vital signs were reasonably stable except for a mild tachycardia with a heart rate of 106 beats/min in the ED. A thorough physical examination showed an inflamed throat with 2 x 2 cm palpable posterior cervical and occipital lymph nodes. An abdominal examination revealed an otherwise soft abdomen with mild discomfort towards the LUQ on deep palpation. The rest of the examination was reasonably benign. Routine investigations showed a mild leukocytosis of 15,000/cm^3^ (4,000-10,000) with predominant lymphocytes, a platelet count of 189,000, a hematocrit of 39.2, a lactic acidosis of 3.4 (0.5-1) mmol/L, a creatinine of 1.2 (0.7-1.2) mL/min, and a mild transaminitis, with an alanine transaminase of 461 units/L and an aspartate transaminase of 228 units/L. Any bowel obstruction was ruled out with the help of an abdominal X-ray. The patient was managed in the ED with intravenous fluids, and a subsequent contrast-enhanced computed tomography scan was done, which demonstrated an enlarged spleen of 18 cm in length and a wedge-shaped hypodense area suggestive of an SI (Figures 1-3). The patient was moved to the in-patient facility to formulate a diagnosis. Several differentials, including viral illnesses, acute hepatitis, and lymphoma, were suspected, for which a wide array of investigations were run to diagnose the disease. A repeat peripheral smear still showed a reactive atypical lymphocytosis without blast cells, but with a mild monocytosis, an elevated erythrocyte sedimentation rate, and C-reactive protein. The rest of the investigations, including lactate dehydrogenase, hepatitis panel, nasopharyngeal swab, and a monospot test, were negative. An EBV serology for IgM was positive, which concluded IM in the patient that led to the splenic infarcts. A conservative treatment approach was taken, and the patient improved in the next few days. He was discharged with instructions to avoid contact sports for three weeks. He returned to the clinic with a complete resolution of his symptoms.

## Discussion

Spleen is an organ of the reticuloendothelial system and a region of blood purification. It has a rich collateral blood supply from the splenic and the short gastric arteries, which is due to the organ's close proximity to the stomach. Nonetheless, it precisely gains 5% of the cardiac output [5,6]. SI can, however, occur despite this adequate blood supply. There are many reasons for this to happen. Many blood disorders can impair the mechanisms of coagulation leading to clots within the splenic substance. This is illustrated by Caremani et al., where they described the incidence of focal splenic lesions ranging from 0.103% to 0.20%, out of which 9.7% infarcts were as a result of occlusion of either the splenic artery or the vein due to thrombosis leading to infarcts within the splenic sinusoids [7]. Of note here are the variety of etiologies that can lead to this complication. These can include pathologies of cardiogenic, vascular, infective, autoimmune, inflammatory, iatrogenic, hematological, oncological, idiopathic, and drug-induced origin. However, the patient had no risk factors of atrial fibrillation or coagulopathies. These were confirmed on an EKG and coagulation markers. As stated earlier, the most common age group for hematological etiologies is less than 40 years, while in the older population, SI is mostly attributed to thromboembolic events [8]. 

On the other hand, infectious causes can also lead to SI. The most common among them is IM, a disease common in the adolescent population. Despite being a self-limiting infection, as is the case with most viral infections, it can lead to dire consequences of infarcts or rupture within the spleen due to the invasion by EBV [9]. As with the rarity of its presentation, the pathophysiology of splenic enlargement secondary to EBV is not yet well understood. Various mechanisms are thought to explain the background of the pathogenesis involved in SI during IM. One such possibility is due to the mismatch in the blood supply to the organ due to hypercellularity secondary to an increased size in acute infections that may be a contributing factor for a localized infarction. It is also proposed that an abrupt rise in the acute phase reactants during the infective phase can impose a transient prothrombotic state with an increased antiphospholipid antibody, lupus anticoagulant, and factor VIII leading to acute infarcts. However, these briefly elevated antibodies do wear off in one to four years and do not carry any significance. Yet another reason could be related to an exponential expansion of B-cells in the acute phase of IM resulting in elevated circulating immune complexes, which can lead to leukocyte aggregation and adhesion, resulting in areas of infarcts. Therefore, there is not a shred of definitive evidence that explains the mechanism by which spleen may get infarcted in cases of IM [10].

Despite a similar presentation in this patient, many differentials emerged, keeping in view the age and a variable presentation. The challenge here was the presence of certain features like a young age, B-symptoms, LAD, splenomegaly, lymphocytosis, and a negative monospot test. These facts were pointing to hematological malignancies, such as lymphoma. A stepwise investigative approach helped to reveal the mystery of this rare disease and its variable presentation. There are essentially many reasons for an extensive diagnostic workup to rule out this disease process. This not only increases the chances of hospital stay but is also not cost-effective. A symptom-focused approach and stepwise limited lab work can aid in reducing these shortcomings. Our case also incorporates the importance of excluding IM in SI as an infective source. This can not only be cost-effective but can also eliminate the need for extensive invasive diagnostic workup. Concerning the rarity of this disease process, an extensive literature search was conducted, and only 25 cases of SI secondary to IM with EBV as an etiological agent were found. However, a total of 13 cases were reported between 2011 and 2018 to the best of our knowledge. All the patients were above 18 years and had no comorbidity yet fell into the catastrophe of SI due to IM. Nonetheless, all the patients were managed conservatively and followed up later with a complete recovery similar to our patient.

## Conclusions

The presented case not only points towards the rarity of SI, as seen in patients with IM, but also highlights the importance of a stepwise approach towards the etiological workup of SI. This, however, aids in the quick diagnosis of the illness and can also decrease the cost of care, hospitalization, and anxiety related to the uncertainty of the diagnosis. In addition to this, our case marks the importance of considering IM in the initial differentials for an infarcted spleen even after a negative monospot test.
